# Time to smell: a cascade model of human olfactory perception based on response-time (RT) measurement

**DOI:** 10.3389/fpsyg.2014.00033

**Published:** 2014-02-04

**Authors:** Jonas K. Olofsson

**Affiliations:** ^1^Gösta Ekman Laboratory, Department of Psychology, Stockholm UniversityStockholm, Sweden; ^2^Swedish Collegium of Advanced StudyUppsala, Sweden

**Keywords:** olfaction, response-time, affect, emotion, valence, object

## Abstract

The timing of olfactory behavioral decisions may provide an important source of information about how the human olfactory-perceptual system is organized. This review integrates results from olfactory response-time (RT) measurements from a perspective of mental chronometry. Based on these findings, a new cascade model of human olfaction is presented. Results show that main perceptual decisions are executed with high accuracy within about 1~s of sniff onset. The cascade model proposes the existence of distinct processing stages within this brief time-window. According to the cascade model, different perceptual features become accessible to the perceiver at different time-points, and the output of earlier processing stages provides the input for later processing stages. The olfactory cascade starts with detecting the odor, which is followed by establishing an odor object. The odor object, in turn, triggers systems for determining odor valence and edibility. Evidence for the cascade model comes from studies showing that RTs for odor valence and edibility assessment are predicted by the shorter RTs needed to establish the odor object. Challenges for future research include innovative task designs for olfactory RT experiments and the integration of the behavioral processing sequence into the underlying cortical processes using complementary RT measures and neuroimaging methods.

## INTRODUCTION

A philosophical thought experiment was recently proposed in which two distinct scents were to be imagined in rapid alteration – an “olfactory trill” (Cooke and Myin, 2011). While it is easy to imagine an auditory trill, as in Chopin’s *Nocturne, Op 62, No 1*, an olfactory percept that rapidly alternates between, say, a woody note and a floral note, is likely unimaginable to even the most sophisticated nose. A starting point for this review is the observation that the temporal resolution of odor perception is limited, at least compared to that of some other senses. The thought experiment described above suggests that the time-scale of the human olfactory system is a defining feature of olfactory experience (Cooke and Myin, 2011).

The current review focuses on evidence from response-time (RT) measures that suggests how important perceptual attributes unfold in time. Little attention has been devoted to integrating findings from olfactory RTs, despite the fact that the first empirical reports were published nearly a century ago (Zwaardemaker, 1925; Wells, 1929). Although several methods may be used to study olfactory processing speed, this review focuses on RTs generated by button-press responses in olfactory tasks that may be completed in one single sniff. The review discusses findings from detection-RT tasks, which typically require a single button-press response, as well as choice-RT tasks, which involve more than one response option and are thus used to study sensory discrimination and cognitive processing. Experiments that are based on subtle odor differences or complex odor mixtures often require evidence accumulation over several sniffs, and are thus outside the scope of the present article (e.g., Bowman et al., 2012).

A key assumption in studies of human information processing is that performance is mediated by a sequence of time-consuming processes, which include perceptually encoding a stimulus, accessing stored information in memory, decision-making, and preparing and executing an appropriate response (Meyer et al., 1988). Attempts to outline mental processes underlying visual cognition have yielded a vast body of literature. It is beyond the scope of this review to give justice to this literature, but it has been reviewed elsewhere (Luce, 1986; Meyer et al., 1988). Despite the challenges of establishing information processing stages in human olfaction, the field has matured enough to begin to integrate findings within a chronometric framework. Below, I provide a brief overview of the role of time in the neurophysiological and perceptual encoding of odors, followed by an overview of studies on human olfactory RTs. I then introduce the cascade model of olfactory perception based on recent evidence. Finally, I discuss theoretical and methodological issues that may be critical for advancing this line of research. A general theme is that methods of studying olfactory RTs are complementary to neuroimaging methods with high spatial resolution but low temporal resolution.

## TEMPORAL ENCODING IN THE OLFACTORY SYSTEM

The issue of time in the neuronal encoding of odors was highlighted in the pioneering works of Maxwell M. Mozell, who discovered that different molecules migrated through the olfactory mucosa with different time-scales. Mozell suggested that “this chronographic differentiation may be one of the mechanisms underlying olfactory discrimination” (Mozell, 1964; Mozell and Jagdowicz, 1973). The time for the transduction of the olfactory stimulus to the nervous system can be approximated to 150 ms (Firestein et al., 1990). These observations have since informed a vast literature on the role of temporal coding in the olfactory system.

Timing is critical for the encoding of odors in the olfactory epithelium and bulb (Schaefer and Margrie, 2007). Studies on several species have shown that olfactory receptor neurons are activated in an odor- and concentration-dependent manner, leading to a temporal pattern of activation that corresponds to distinct stimulus features. Principal neurons in the olfactory bulbs of vertebrates, and the homologous antennal lobes of insects, respond to odor input by changing the timing and frequency of neuronal spikes; these temporal changes enable the behavioral discrimination of distinct odors through a variety of mechanisms (Laurent, 2002). Neurons in the immediate downstream projection sites of the principle neurons (e.g., the piriform cortex in vertebrates; mushroom bodies in insects) read out a converging afferent input that evolves over the time-scale of a sniff. Recent findings from optogenetic imaging in mice suggest that in the mammalian olfactory system, the temporally defined output from the olfactory bulb is translated into a spatial ensemble code at the level of the piriform cortex. The piriform cortex may thus act as a “sequence detector” of output from the olfactory bulb (Haddad et al., 2013). Neurons in the piriform cortex further propagate sparse signals that are highly specific to particular odors. The rapid evolution of an olfactory percept within the sniff cycle is critical for adaptive behavior. Schaefer and Margrie (2007) illustrated the importance of time in odor discrimination by evoking the metaphor of two jigsaw puzzles. When the jigsaw puzzles depict two very similar pictures, many pieces have to fall in place before it may be determined that the pictures are different. When the jigsaw puzzle pictures are very different, only one or a few pieces are needed to make this decision. Analogously, two very similar odors gradually activate glomeruli in the olfactory bulb over the course of a sniff, and a reliable behavioral discrimination may only be established when most or all of these glomeruli have become activated. In contrast, two very different odors may be discriminated early in the evolution of glomeruli activation. It has been suggested that latency patterns of output from the olfactory bulb may contain the most critical piece of information needed for higher brain centers to identify odors (Junek et al., 2010).

### OLFACTORY OBJECT PERCEPTION

It is widely agreed that the underlying molecular features of an odor are not directly accessible to conscious experience (e.g., Wilson and Stevenson, 2003). The neural mechanisms reviewed above suggest instead that the “qualia” of human olfactory sensations likely evolve with the time-scale needed for the olfactory cortex to determine the unique spatiotemporal firing properties elicited by a particular odor stimulus. But bottom-up mechanisms are not enough to explain perceptual representations of odors – instead, the neural response to the multiple molecular features of a smell is thought to be synthesized into a unified percept, which is commonly referred to as an olfactory object (Wilson and Stevenson, 2006). These objects are not only the products of molecular features, but also of perceptual memory; odor inputs are matched to perceptual object templates (“engrams”) that have been established at prior exposure, and a good match produces a subjective feeling that the odor is familiar (Stevenson and Boakes, 2003). When a chemical component is added to or subtracted from a complex but familiar odor mixture, neurons in the piriform cortex retain a uniform response (Barnes et al., 2008). Such neuronal mechanisms of pattern completion and pattern differentiation help to maintain stable representations of odor qualities and enable odor recognition despite stimulus variability. Olfactory object perception thus resembles visual object perception, but unlike the visual system, olfaction has a limited capacity to process multiple objects in parallel. Instead, molecular odor features interact to produce a unique perceptual quality (Snitz et al., 2013). Although the ability to dissociate odor features may be enhanced somewhat through training, most individuals are not capable of distinguishing three or more familiar odors when they are presented in a mixture (Livermore and Laing, 1996). In humans, this synthetic processing that translates odor features to objects likely takes place in areas such as the piriform cortex (Gottfried, 2010) and the orbitofrontal cortex (Li et al., 2006). The olfactory object-system might modify perceptual odor objects even after only a few minutes of exposure (Li et al., 2006). However, highly familiar odors correspond to well-established odor templates, and using such odors may bring out the full benefits of object-based perception. The overlap between an odor quality and a corresponding template may be empirically established by having participants rate an odor’s “perceptual quality,” that is, how well a given odor (e.g., rose essential oil) represents an olfactory template (indicated by the label “rose”).

## BEHAVIORAL RESPONSE-TIMES TO ODORS

### OLFACTION AND MENTAL CHRONOMETRY

The use of response latencies as a measure of physiological events in the nervous system was the invention of Helmholtz, who already in the 1850s designed the simple-response time design in which a participant presses a button upon sensing a stimulus (Helmholtz, 1883). Helmholtz showed convincingly that mental processes and their neural underpinnings involve time-consuming events, and paved the way for further chronometric investigation of mental phenomena. Not long thereafter, Donders (1868/1969) invented the binary choice-response time paradigm for rapid stimulus evaluations, which allowed the researcher to address more elaborate psychological questions. Donders also introduced the subtraction method to establish the time-scale of underlying component processes; for example, the speed difference between the time needed to classify a stimulus compared to the time needed to simply detect its presence defines the temporal extent of the “classification” processing stage. Pioneers such as Moldenhauer (1883) and Zwaardemaker (1895) invented olfactory stimulation techniques that paved the way for applying the basic chronometric paradigm to human olfaction.

An early use of olfactory RTs in psychology is a study by Wells (1929), where participants rapidly classified odors as being either pleasant or unpleasant. The results showed that these evaluations of odor valences could be accurately produced with button-press RTs within about 0.9 s of the sniff onset. This result provided evidence that such emotional responses could not be the result of the brains interpretation of a peripheral bodily response, as had been suggested by William James and Carl Lange. Instead, olfactory valences were rapidly and directly produced by the brain.

### TEMPORAL INTEGRATION IS PRESENT IN HUMAN OLFACTION

Early research on human olfactory perception established that faint odors were detected more reliably when participants sampled the odor with vigorous sniffs rather than with longer sniffs (Le Magnen, 1945). This finding suggested that odor-evoked activity is integrated over a time-window to generate a signal average, and that the amplitude of this signal determines whether the odor reaches consciousness. This temporal averaging in human olfaction appears to plateau within 500 ms of odor sampling at “natural” sniff velocities. Further increased sampling does not influence odor thresholds or perceived odor intensities. The plateau is thus well-established within a normal sniff, which lasts for about 1.6 s (Laing, 1983, 1985).

### ODOR VALENCE AND OVERALL PROCESSING SPEED

A long-standing issue in human olfactory perception is whether certain classes of odors enjoy the privilege of “early access” to the perceptual system due to their great adaptive relevance (see e.g., Doty, 2010). As humans readily evaluate odors based on their valence, and unpleasant odors may be harmful to the organism, it may be hypothesized that unpleasant odors are detected faster than pleasant odors. This hypothesis is congruent with the notion that valence might be rapidly decoded by the brain at the earliest processing levels, and that the neural response to odor valence provides input to all further sensory analysis (Yeshurun and Sobel, 2010). The early study by Wells (1929) found that detection-RTs were the same for pleasant and unpleasant odors. Since that early negative finding, a few studies have directly compared the latencies of detection-RTs and choice-RTs for unpleasant and pleasant odors. In a study using unirhinal odor stimulation, Bensafi et al. (2001) found that unpleasant odors yielded shorter RTs than pleasant odors. However, this effect was only present during valence assessment, and not during detection, familiarity, or intensity assessments. Moreover, the effect was only seen for right-nostril stimulation, and not for left-nostril stimulation. Similar results followed from an experiment with odorants lacking trigeminal effects (vanillin and indole; Bensafi et al., 2002). Since the olfactory nerve projects ipsilaterally to downstream sites, the authors interpreted their findings within a framework of emotional lateralization, and concluded that emotional processing of unpleasant odors was emphasized in the right hemisphere. A further experiment found that unpleasant odors were processed faster than pleasant odors, but again, this effect was only present in valence evaluations and not in detection, intensity, or familiarity assessments (Bensafi et al., 2003). While the results from these studies show that odor valence is lateralized, they do not show that unpleasant odors have “early access” to the olfactory-perceptual system, as this would have resulted in faster RTs independent of task and nostril. Instead, the results are compatible with the notion that odor valence was determined downstream, as the lateralized effects of valence did not carry over to other tasks, as would have been the case if odor valence evaluation was an early and mandatory stage in a causal processing chain.

More recent studies have focused on odor detection-RTs but have provided mixed results. One study reported shorter RTs for an unpleasant odor compared to a pleasant odor over a range of concentrations (valeric acid and isoamyl acetate; Jacob and Wang, 2006). Another study investigated RTs in a detection task for four different odors that represented variation in both perceived valence (high/low) and edibility (edible/inedible; Boesveldt et al., 2010). The results showed that an unpleasant odor from an edible source (resembling fish) was the most rapidly detected within the odor set. Among odors from inedible sources, there were no differences according to valence. While the authors expressed caution because data were only obtained for one odor per category, it was proposed that humans might have evolved a mechanism for the rapid detection of ecologically relevant food odors that warn of potential danger (i.e., unpleasant odors emanating from edible sources). However, a recent study did not find such RT differences between a pleasant and an unpleasant food-related odor in a detection task (La Buissonniere-Ariza et al., 2013). In our studies on RTs for a wider range of odors, odor valence is not correlated with RTs in detection, identification, valence, or edibility decision tasks, whether the odor is from edible or inedible sources (unpublished observations).

The results reviewed above have not provided ubiquitous support for the notion that odors are encoded differently depending on their valence. In future studies, certain methodological aspects should be considered. It is unclear whether task-related differences (e.g., simple detection-RT versus binary choice-RT) have contributed to the inconsistent pattern of results. Most previous studies did not include assessments of how familiar the odors were to the participants or how well the odors matched familiar object templates, but such variables may influence olfactory RTs. For future investigation, it might be hypothesized that if the human brain had evolved a particular system for rapidly encoding intrinsically unpleasant odors (assuming such odors exist), this processing advantage is likely to be stronger for complex natural odors that are often encountered in the environment rather than unfamiliar monomolecular odors, and to manifest in detection-RT tasks as well as in downstream processes assessed with choice-RTs, such as classifications of valence and edibility and matching odors to labels or pictures. These criteria have not yet been met. In fact, while particular odors may be more rapidly detected than others based on a variety of stimulus factors, available evidence from olfactory RTs suggests that odors may not be encoded differently depending on their valence.

## A CASCADE MODEL OF HUMAN OLFACTORY PERCEPTION BASED ON CHOICE-RTs

The idea at the center of this review is that the speed of olfactory decision-making, assessed with choice-RTs, may be theoretically informative as to the fundamental psychological processing of odors. The flow of information within the olfactory system upon sensory input may be described as a cascade; a causal chain of rapidly unfolding perceptual features. RT measurements may be particularly useful to map this olfactory cascade. Of particular relevance to this discussion are theories that assume radically different activation sequences of olfactory-perceptual features (Wilson and Stevenson, 2003, 2006; Yeshurun and Sobel, 2010). According to the object-centered account, odor objects are constructed early in the processing sequence. The result of the object processing feeds into other systems to determine valence, edibility, and other important attributes, based on previous experiences with the odor object. In contrast, the valence-centered approach assumes that odor valence is determined by molecular stimulus features (Khan et al., 2007). Through evolution, mammals developed mechanisms by which the intrinsic odor valence is rapidly and effectively decoded (Yeshurun and Sobel, 2010). The stimulus-driven, intrinsic valence of odors might also be decoded from cortical processing patterns (Haddad et al., 2011). According to this view, semantic analysis of odors is impaired relative to the visual system because odor names and identities have to be reconstructed from their unique valences, which is a very difficult task (Yeshurun and Sobel, 2010).

Below, I present a cascade model that offers a chronometric approach to understand human olfactory perception. It emphasizes the succession of processing stages that unfold when we encounter a recognizable odor. This model is based on recent RT evidence from odor detection, object categorization, label matching, valence, and edibility evaluation tasks that reveal how different features of the olfactory percept unfold in time. These studies involve binary choice-response tasks in which the odor set, sniffing behavior, and button-press response format were the same for all odor tasks. The RT differences between tasks are therefore assumed to roughly reflect the time required for the task-specific olfactory-perceptual computations. The principal features of the cascade model are reviewed below.

### EARLY ACTIVATION OF ODOR OBJECTS

A key assumption of the cascade model is that differences in RTs across tasks are indicative of processing stages within the olfactory cascade. Tasks that are faster to carry out for a given set of odors engage in earlier processing stages than tasks that require longer processing times. The first stage of odor processing is detecting odors, regardless of their quality. We have found that in a sequence including 50% blank trials and 50% odor trials of varying odors, detection takes about 800 ms from the onset of the sniff-cue to an accurate button-press response that confirms an odor is present (Olofsson et al., 2013a). Previous studies have yielded similar RTs (Wells, 1929; Laing, 1986; Laing and MacLeod, 1992).

The task of odor matching to labels is a method of probing access to odor objects, and objects may be established early in the processing sequence (Stevenson and Boakes, 2003; Wilson and Stevenson, 2006). In the matching task, a label is presented prior to the odor; when the odor is released, participants indicate whether it is congruent or incongruent with the label. As predicted from the object-based account, odor matching to labels was executed with near-ceiling level accuracy at about 1000 ms after sniff onset (Olofsson et al., 2013a). This result suggests that an odor object is established at about 200 ms following detection. Evaluations of valence (rapidly choosing whether the odor was pleasant or unpleasant) and edibility (rapidly choosing whether the odor came from an edible or inedible source) were carried out at a slower speed of around 1100–1200 ms. Binary evaluations of odor valence and edibility were significantly slower than the evaluations of odor objects. Follow-up analyses confirmed that odor valence and edibility RTs were not affected by odors that were ambiguous in their valence or edibility, which could have prolonged RTs. The RT advantage of odor object processing before valence processing was also found in a recent study that used a different task design (Olofsson et al., 2012). In that study, two categorization tasks were constructed – one requiring access to objects and one requiring access to valences – in which two odors were delivered on two consecutive sniffs. The object-task was to classify the second odor as belonging to either the same object category or a different category as the first (odors belonged to one of four categories: floral, fuel, mint, and fish). The valence task was to determine whether the second odor was more or less pleasant than the first. Results showed slower RTs and plenty of internal inconsistencies in the valence task compared to the object category task, consistent with the object-based approach to olfactory perception. Even when omitting “difficult” trials in the valence task (i.e., those that included two similar odors) from analysis in order to achieve similar accuracy rates across tasks, responses were slower in the valence task (Olofsson et al., 2012). The results suggest that odor objects are processed before valence and edibility evaluations in the olfactory system.

### OBJECT-LEVEL SEMANTIC PRIMING

Semantic priming is a facilitation of stimulus processing that is based on extracting the meaning of a prior stimulus. By using verbal cues to prime odor-based decisions, information may be gained about the olfactory system. When the trial structure includes a verbal cue (e.g., “orange”) that is followed by an odor that is either matching or non-matching to the cue, choice-RTs to the odor are decreased when odors are matching (orange odor) compared to non-matching (other odors) (Olofsson et al., 2012). Odor processing is thus facilitated by the information provided by the cue. In contrast, cues that provide categorical information about the odor valence (e.g., “pleasant”) do not facilitate processing speed for congruent odors (e.g., orange odor is regarded as pleasant). This supports the notion that specific object templates may be pre-activated by a verbal cue at an early-stage of processing (Stevenson and Boakes, 2003). Similarly, functional magnetic resonance imaging (fMRI) results suggest that a cue that instructs the participant to focus on an odor in a binary mixture leads to a “pre-activation” of a neural pattern of activity in the piriform cortex prior to the odor delivery; this pattern resembles the pattern of activity that is activated by the odor itself (Zelano et al., 2011). This result and other findings from pattern-analyses of fMRI data (Howard et al., 2009) suggest that templates for odor objects and categories are encoded as distributed patterns of activity in the human piriform cortex.

As it was proposed that odor valence is encoded at the earliest perceptual stages (Yeshurun and Sobel, 2010), we investigated whether valence similarity between the label and the odor would cause semantic interference in the priming task. It was predicted from the valence-based approach that when the label of a pleasant odor (e.g., the word “lemon”) was followed by a similarly pleasant odor (e.g., the odor of rose), participants would need more time to decide that the trial was incongruent because these odors have similar valence, but when the label “fish” was followed by the odor of rose the RTs would be shorter. However, there was no such interference from valence on the odor object processing speed (Olofsson et al., 2012). In sum, semantic priming was only effective at the level of odor objects. These results indicate that valence does not affect semantic analysis at the early object stage, but support the notion that odor valences are computed after odor objects.

### CAUSAL RELATIONSHIPS AMONG PROCESSING STEPS

**FIGURE 1:**
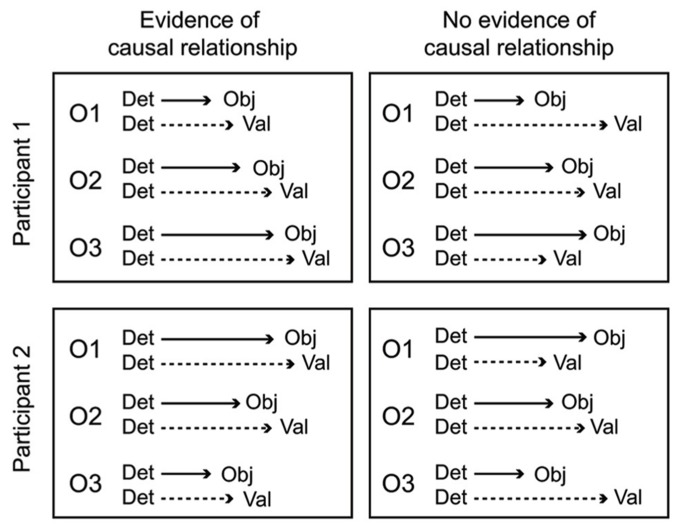
**Hypothetical RT outcomes in one case of causal mediation of odor valences by objects (left panel), and one case of no causal relationship between object processing and valence processing (right panel).** In both cases, data from three odors (O1, O2, O3) have been aligned to the time of odor detection, a starting point for further conscious processing. A causal relationship between odor objects and valences is inferred in the left panel because odor valence decisions are executed at a predictable lag following object decisions. However, in the right panel these decisions are uncorrelated, and completely independent processing pathways cannot be refuted. The causal relationship in the left panel holds for both hypothetical participants, even though the olfactory processing speed is fastest for O1 in participant 1, but fastest for O3 in participant 2.

**FIGURE 2:**
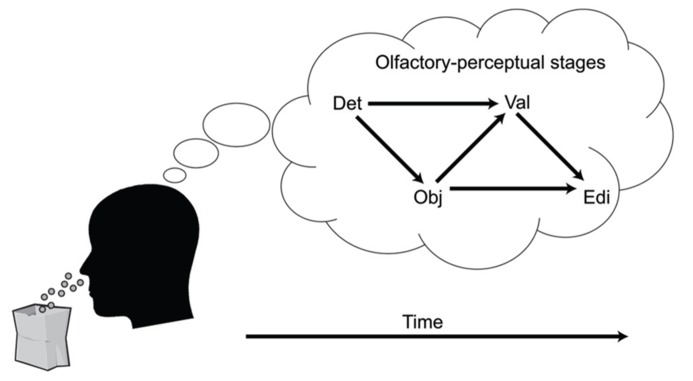
**FIGURE 2. A cascade model of olfactory perception.** The figure proposes four stages of perception that unfold upon an olfactory input: Detection (DET), Object processing (OBJ), Valence (VAL), and Edibility (EDI). Decisions that require processing at later processing stages are mediated by processing at earlier levels.

A key aspect of the cascade model is that different processing stages are causally related (i.e., at least partly “serial” rather than completely “parallel” processing), and that these relationships manifest as temporal contingencies that can be measured with RTs. As causal relationships cannot be directly observed, they must be inferred from temporal covariation. **Figure 1** illustrates hypothetical RT outcomes in a case where there is a causal relationship between these constructs, and a case where there is no such relationship. We used regression-based analyses to model the assumed causal relationship between odor detection, odor object identity, odor valence, and edibility (Olofsson et al., 2012, 2013a). The hypothesis, derived from the object-based approach, was that the time needed to conduct a perceptual decision about the valence or edibility of a given odor would be systematically delayed by the time it took to establish its perceptual object. Although object-based RTs for a given odor may differ across individuals, the cascade model assumes a systematic prolongation of valence and edibility decision times for the individual. In our first experiment (Olofsson et al., 2012), we were able to successfully predict valence-based categorization RTs from the more rapid object-based categorization RTs using a regression approach. The effect occurred after controlling for differences related to participants, as well as stimulus factors. This result suggested not only that objects were established before valences, but objects also appeared to trigger valences in a causal way. In a subsequent experiment (Olofsson et al., 2013a), we further supported this notion of causality using mediation analysis (Preacher and Hayes, 2004). We modeled the pathway from odor detection to valence and edibility via odor object identification as a mediating variable. We constructed a mediation model of the RT data that included a “direct” processing route from detection (the predictor) to valence (the outcome), and an “indirect” route linking detection and valence by way of identification (the mediator). By determining the significance of the direct and indirect routes, the model tested whether the relationships between our key variables would conform to either parallel routes (no mediation), a serial (mediated) route, or dual-processing routes (both mediated and unmediated). Results indicated dual-processing routes to odor valence; object-mediation played a significant role in processing odor valence, but there was also information that bypassed objects. When replacing valence RTs with edibility RTs as outcomes, the result suggested a complete mediation of edibility by odor objects (serial route). However, when adding valence as a second mediator to determine olfactory edibility RTs, the explained variance of edibility RTs increased further. That result suggested that the valence RT for an odor helps to determine its edibility RT. Thus, in two experiments we showed that not only were object decisions faster than valence and edibility decisions, but that there also appeared to be a causal connection such that objects trigger valence and edibility evaluations. The results from RTs in the different olfactory tasks, combined with the results from mediation analysis, are summarized in an illustration of the cascade model (**Figure 2**). The figure shows how different processing steps are activated at different time-points upon the receipt of an odor and which processing routes are activated to complete a given task. When a decision is made, the corresponding motor response is executed. As shown in **Figure 2**, the processes unfold at different time-points and are causal in that earlier processes mediate later processes. The pathways in the figure are inter-connected, which is supported by the result that the relatively long RTs of odor edibility are better predicted from a combination of the shorter RTs of several upstream processes.

## FUTURE DIRECTIONS

Although human olfaction may be regarded as a slow and imprecise sensory system, studies of olfactory RTs show that untrained participants are able to carry out rapid olfactory-based decision-making. In fact, after subtracting the time needed for sniff onset (about 200 ms; Olofsson et al., 2012), chemical interactions with the olfactory receptor neurons (about 150 ms; Firestein et al., 1990), and the time required to execute a behavioral response on a visual cue without olfactory involvement (about 300 ms; Brown and Heathcote, 2008), major olfactory decisions may be confined to a rapidly unfolding cascade of inter-connected processing steps within a 500 ms interval. Within this interval, recent results suggest that odor detection is followed by the establishment of an odor object, which in turn is used to activate valence and edibility evaluation systems (Olofsson et al., 2012, 2013a), an outcome that aligns with the object-based approach to olfactory perception (Wilson and Stevenson, 2006). Challenges for future research are discussed below.

### CONCEPTUAL ISSUES

A key challenge for future research is to develop new experimental designs by which to assess the sequence of processing stages in the human olfactory cascade. It should be noted that there is not yet a consensus on what the major olfactory processing features are, or how they should be measured in an unbiased way. Although the available evidence suggests that odor objects exist, that they are established early in the processing stream, and that they causally mediate responses in other perceptual tasks, the sequence proposed in the cascade model needs to be further validated across different task designs. Insights into how different operationalizations affect olfactory processing speed will minimize the risk of bias. For example, it may be assumed that valence is a continuous construct whereas the odor objects (or functional categories) are binary constructs. Thus, a binary assessment of odor valence in previous studies might have put the valence dimension at an operational disadvantage. However, this operationalization was motivated by theoretical concerns. According to the valence-based approach, continuous valence encoding is the starting point for olfactory processing (Yeshurun and Sobel, 2010), including any binary categorization into floral, fuel, mint, or fish odor groups, or deciding whether an odor matches the label “lemon.” Such object evaluations are effortfully achieved only after the exact valence of an odor is decoded. Therefore, under the assumption of the valence-based approach, it should be impossible for participants to complete an object-categorization task faster than a valence-categorization task. However, as reviewed above, such results emerged consistently (Olofsson et al., 2012, 2013a). If, on the other hand, this assumption is abandoned for a view that odor objects and object categories are binary constructs, but valences are continuous constructs, there is still a possibility that odor valences may in fact be processed early, even though this early processing did not manifest in short RTs in previous studies. However, available data do not support the notion that valence processing speeds were at a disadvantage because of the binary tasks used in previous studies (Olofsson et al., 2012, 2013a); for example, odors of ambiguous valence (e.g., garlic odor was often evaluated by participants as mildly unpleasant) did not generate slower RTs than more extremely valenced odors such as fish (Olofsson et al., 2013a). However, binary valence decisions (e.g., indicating that an unpleasant fish odor was unpleasant) were slower and less accurate than object decisions for the same odor. This finding appears difficult to reconcile with any model in which valence is processed faster than objects. Instead, the available evidence from detection-RTs and choice-RTs indicates that odor valence is not encoded at the earliest stage of odor perception, but is instead slower and more inconsistent than evaluations related to odor objects. While previous studies were designed to assess predictions from the object-based and valence-based approaches to olfaction, future studies should not be constrained by these specific theories and instead focus on constructing novel task designs that might provide further converging evidence as to how the olfactory cascade unfolds over time.

### INTEGRATION OF RESULTS FROM RTs AND CORTICAL METHODS

The cascade model of human olfaction suggests that RT measures of olfaction may provide a new source of information into the hierarchical nature of the olfactory system. The idea that olfactory processes are organized in a hierarchical fashion is not new, but it has until now been supported mainly by lesion data (e.g., Zatorre and Jones-Gotman, 1991; Olofsson et al., 2013b) and functional neuroimaging data (e.g., Savic et al., 2000). As the olfactory system is characterized by extensive feedback loops between higher and lower centers, functional neuroimaging methods may not easily dissociate “early-stage” activation from recurrent activation through feedback from downstream centers. Structural neuroimaging techniques may be used successfully in groups with focal neurological damage to reveal hierarchical functions within the olfactory system. However, compensation and reorganization effects following a lesion preclude definitive conclusions (Rorden and Karnath, 2004). The olfactory cascade model assumes that tasks that produce shorter RTs are computed upstream from tasks that produce longer RTs, and that a temporal contingency between these RTs required to carry out two different tasks (e.g., establishing objects and valences) on an odor-by-odor basis is evidence of a causal link between the mental operations required to carry out the tasks. The logic underlying the olfactory cascade model makes it complementary to functional and structural neuroimaging methods.

Event-related potentials (ERPs) might provide a means to monitor the rapid unfolding of olfactory-cognitive processing stages in real-time. So far, there has been little theoretical integration of results from olfactory ERPs and results from RTs. Perhaps this is partly due to the fact that effects of stimulus intensity and individual differences on ERPs are profound and may overshadow more subtle cognitive processing signatures (e.g., Hummel et al., 1998; Olofsson and Nordin, 2004; Nordin et al., 2005; Olofsson et al., 2005). From the perspective of the olfactory cascade model, semantic priming paradigms may be particularly suitable to investigate ERP responses in olfactory decision-making. In semantic priming paradigms, the ERP differentiates between semantically incongruent and congruent targets in a negative deflection known as the N400 effect (Kutas and Hillyard, 1980; Kutas and Federmeier, 2011). Only a few studies have used odor targets to elicit an N400 component (e.g., Grigor et al., 1999; Safarzi et al., 1999; Kowalewski and Murphy, 2012). Little is yet known about the exact timing and neural generators of the olfactory N400. But future investigations might integrate ERPs with behavioral RT paradigms to probe the neural correlates of the olfactory cascade as it unfolds in real-time.

## CONCLUSION

Time is a critical feature of the neural processing of odors in the olfactory epithelium and bulb. This review shows that time might also be essential for understanding the psychological processing of odors. The temporal resolution of olfactory perception appears to be confined within the time limits of a sniff. As our system for conscious odor perception likely co-evolved with the respiratory system by which we sample odors, our failure to imagine high-frequency “olfactory trills” might be expected. Despite these constraints, recent studies that measure RTs for odors reveal that several perceptual processing stages unfold in a cascade-like manner already within the first second following sniff onset. These findings provide the basis for a cascade model of human olfactory perception. According to the cascade model, odors are encoded through distinct processing steps that are causally related: detection, object, valence, and edibility. For a given odor and individual, the time needed to establish the odor object will influence the time needed to establish valence and edibility decisions downstream. Challenges for future studies include the invention of novel task designs to assess key olfactory processing features, and the integration of behavioral results with assessments of cortical processing. Such developments will further elucidate how odor evaluations rapidly unfold as a causal sequence of mental operations within the time-course of a sniff.

## Conflict of Interest Statement

The author declares that the research was conducted in the absence of any commercial or financial relationships that could be construed as a potential conflict of interest.
